# Ecosystem service lens reveals diverse community values of small-scale fisheries

**DOI:** 10.1007/s13280-020-01405-w

**Published:** 2020-11-03

**Authors:** Kara E. Pellowe, Heather M. Leslie

**Affiliations:** 1 Stockholm Resilience Centre, Stockholm University, Kräftriket 2B, 106 91 Stockholm, Sweden; 2 Darling Marine Center, University of Maine, 193 Clarks Cove Road, Walpole, ME 04573 USA; 3 School of Marine Sciences, University of Maine, Orono, MA 04469 USA

**Keywords:** Community value, Cultural ecosystem services, Cultural keystone species, Ecosystem services, Gulf of california, Small-scale fisheries

## Abstract

The ocean provides benefits to coastal communities around the world, however, the depth and complexity of people’s interactions with marine ecosystems are not well represented in many marine management initiatives. Many fisheries are managed to maximize provisioning value, which is readily quantified, while ignoring cultural values. An ecosystem services approach that includes both provisioning and cultural services will enable managers to better account for the diverse values marine fisheries provide to coastal communities. In this study, we assess community values related to a top fished species, the Mexican chocolate clam, *Megapitaria squalida*, in Loreto, Baja California Sur, Mexico. We conducted an exploratory analysis based on 42 household surveys, and found that community members perceive multiple provisioning and cultural benefits from the clam, including community economic, historical, and identity values. Despite reporting infrequent harvest and consumption of clams, participants perceive the species as an important part of community identity, highlighting the role of Mexican chocolate clams as a cultural keystone species in the Loreto region. Fisheries management that recognizes the full range of ecosystem services a species contributes to coastal communities will be better equipped to sustain these diverse values into the future.

**Electronic supplementary material:**

The online version of this article (10.1007/s13280-020-01405-w) contains supplementary material, which is available to authorized users.

## Introduction

The ocean provides many benefits to coastal communities, including food, income, recreational opportunities, and aesthetic values (Halpern et al. 2012; Loomis and Paterson 2014), yet the depth and complexity of interactions between people and marine ecosystems are not well understood (Villasante et al. 2013). Management of fisheries and decisions related to governance of marine ecosystems reflect society’s values, priorities, and desires for ecosystems to produce certain benefits. These decisions are complicated by multiple and sometimes contradictory goals, with priority often given to values that can be readily quantified in economic terms (Loomis and Paterson 2014). A holistic understanding of the values produced by marine ecosystems is necessary, if management is to accurately reflect diverse values and balance trade-offs between alternate priorities.

Full consideration of the values associated with ecosystem services will better enable resource managers to address the needs and perspectives of different stakeholders (Chan et al. 2012b). Ecosystem services are the benefits that an ecosystem provides to people (Millennium Ecosystem Assessment 2005). The Millennium Ecosystem Assessment (2005) outlined four categories of ecosystem services: supporting—those services that make it possible for ecosystems to continue providing the other three types of services (e.g., primary production); provisioning—products obtained from ecosystems (e.g., food); regulating—benefits produced through ecological processes (e.g., water purification); and cultural—nonmaterial benefits of ecosystems (e.g., recreation and sense of place). The ecosystem services approach is a useful tool for understanding the connections between humans and ecosystems that goes beyond quantifiable outcomes such as income and food provision to include cultural and social values (Chan et al. 2012b). Earlier work on ecosystem services involved the integration of biophysical and economic perspectives to assess the value of biophysical processes in economic terms (Daily et al. 2000; Turner and Daily 2008). Economic approaches have been useful in advancing understanding of human–nature relationships and facilitating integration of ecosystem-related values into decision-making (Turner and Daily 2008). However, economic approaches fail to encompass dimensions of value that cannot be quantified in economic terms, including many cultural and non-use values (Chan et al. 2011, 2012b). Resource management that is focused on a limited set of ecosystem services may lead to unexpected regime shifts and sudden losses of other ecosystem services (Gordon et al. 2008; Bennett et al. 2009). A growing body of literature highlights the importance of considering and assessing cultural ecosystem services, in addition to provisioning services (Martín-López et al. 2012, 2013; Hernández-Morcillo et al. 2013; Oteros-Rozas et al. 2014; Dickinson and Hobbs 2017). Cataloguing the complete suite of values marine ecosystems produce is a crucial step in managing in a way that both protects crucial benefits and better attends to trade-offs among the diverse values and priorities of coastal communities (Loomis and Paterson 2014).

Provisioning services, such as clean water, food, and income, are essential for providing the basic necessities of life, maintaining security, and protecting human health (Millennium Ecosystem Assessment 2005). As a country follows a development trajectory, human dependence on provisioning services tends to decrease, while dependence on cultural ecosystem services increases (Guo et al. 2010). Unlike provisioning services, which may be replaced by technical innovation or trade as they are degraded, cultural services are not as readily replaced (Millennium Ecosystem Assessment 2005). Cultural ecosystem services are more likely to be co-produced through the interactions between people and their environment, resulting in a tight coupling between the cultural benefits of ecosystems and people’s held values and preferences (Russell et al. 2013; Dickinson and Hobbs 2017). Cultural services are also reflective of people’s environmental decision-making (Martín-López et al. 2013), and can improve human health and well-being through personal and community connections to natural systems (Russell et al. 2013). Thus, it is critically important to account for and assess both provisioning and cultural values if marine management is to preserve both the basic necessities of life provided by fisheries, as well as sociocultural value that connects people and the sea.

In fisheries, resource exploitation by humans can significantly affect system structure and functioning, and impact the long-term sustainability of human–resource interactions (Basurto et al. 2013; Partelow and Boda 2015). Fisheries provide many valuable benefits to coastal communities, yet their sustainability is threatened by overexploitation, pollution, and environmental variability, among other stressors (Béné 2006; Halpern et al. 2012). Traditional fisheries management focuses primarily on fisheries yield as a product of ecological processes and driver of economic benefits, and has come a long way in acknowledging and understanding the heterogeneity of ecological systems. However, a parallel understanding of variety within social systems is often missing (St. Martin et al. 2007). Given the deep and complex ways in which people interact with marine ecosystems (Villasante et al. 2013), particularly through fisheries, the focus of traditional fisheries management is too narrow and overlooks the many other ways in which people interact with and derive benefits from marine species and ecosystems. Meeting the challenge of fisheries management requires moving beyond assessments solely of environmental variables and species interactions to develop a better understanding of sociocultural values and local knowledge of coastal communities and fishers (St. Martin et al. 2007; Johnson 2018; Smith and Basurto 2019).

For small-scale fisheries, our very definitions, typically centered on technology and harvest, ignore the sociocultural characteristics of these fisheries that set them apart from other types of fishing (Smith and Basurto 2019). An ecosystem services approach can illuminate important connections between people and nature and help untangle complex interactions shaping small-scale fishery systems. On the Gulf of California coast of Baja California Sur, Mexico, the Town of Loreto relies on fishing and tourism to support the local economy. These activities are primarily focused on the marine park that the town hosts, Loreto Bay National Park. The national park is home to many species, including the Mexican chocolate clam, *Megapitaria squalida*. The clam is one of the top species harvested by biomass in Loreto (Pellowe and Leslie 2017), and is a local culinary specialty with a rich history of use. As is the case for many fished species, fisheries management of Mexican chocolate clams in Loreto Bay National Park focuses on the maximization of fisheries and economic yield. However, based on the importance of Mexican chocolate clams to local livelihoods (Pellowe and Leslie 2019), we hypothesize that the relationship between people and Mexican chocolate clams in the Loreto region is more multi-dimensional than is currently captured by fisheries management.

This exploratory study presents a novel approach for assessing community values of a single fished species. Using household surveys, this study elicits data on the suite of ecosystem services provided by Mexican chocolate clams to households in this region, using a set of values adapted from previous studies of ecosystem services (Rolston and Coufal 1991; Reed and Brown 2003; Millennium Ecosystem Assessment 2005; Raymond and Brown 2006). In addition to assessing the range of provisioning and cultural values that Mexican chocolate clams provide to households in Loreto, we also assess community perceptions of change related to the clams, since perceptions of change shape people’s environmental decision-making and can help to illuminate priorities for management (Gobster et al. 2007). Finally, we explore how fishery management might better account for trade-offs among varied community values and priorities.

## Materials and methods

### Location of study

**Fig. 1:**
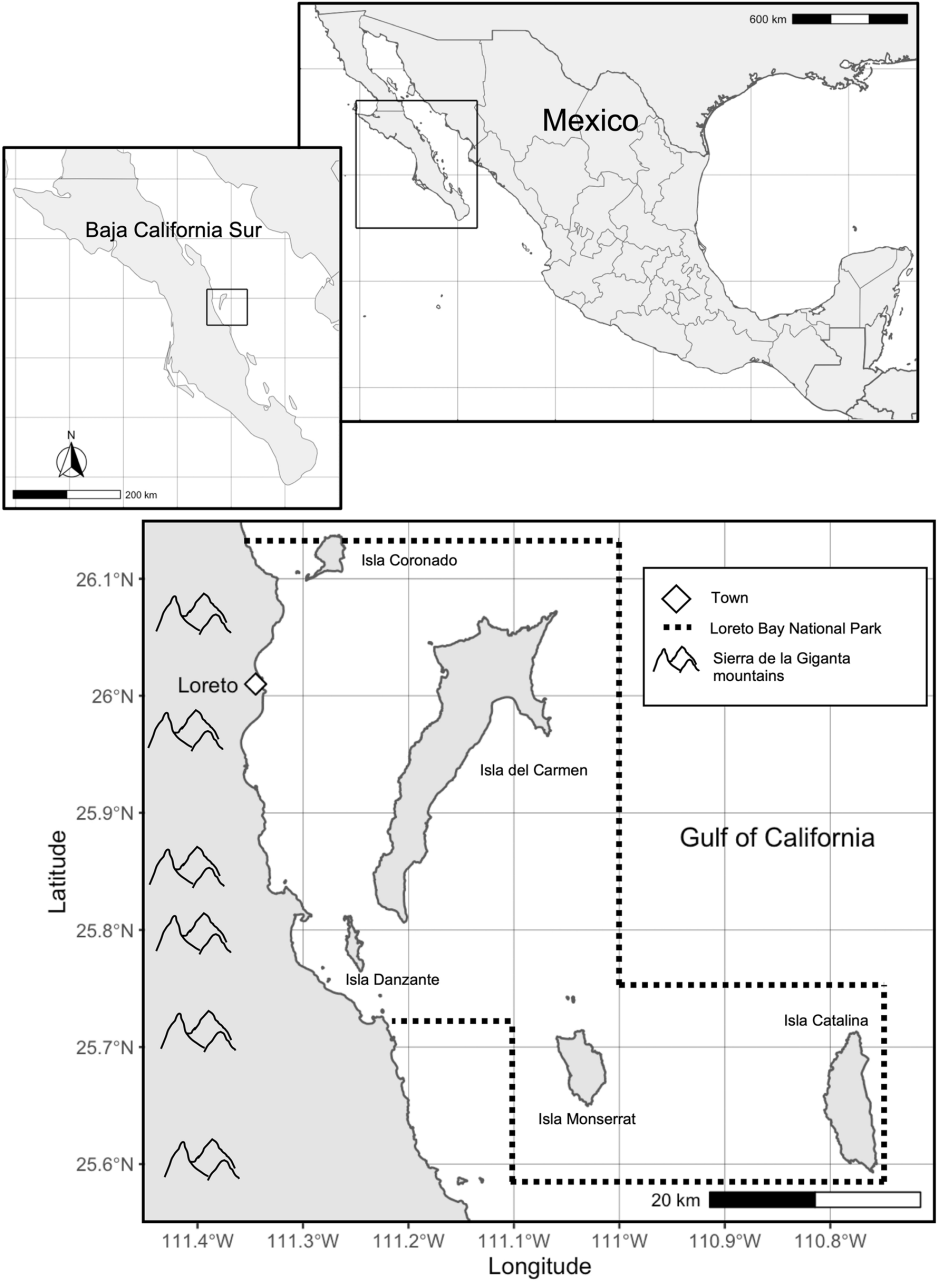
Map of Loreto Bay National Park, Baja California Sur, Mexico

**Fig. 2:**
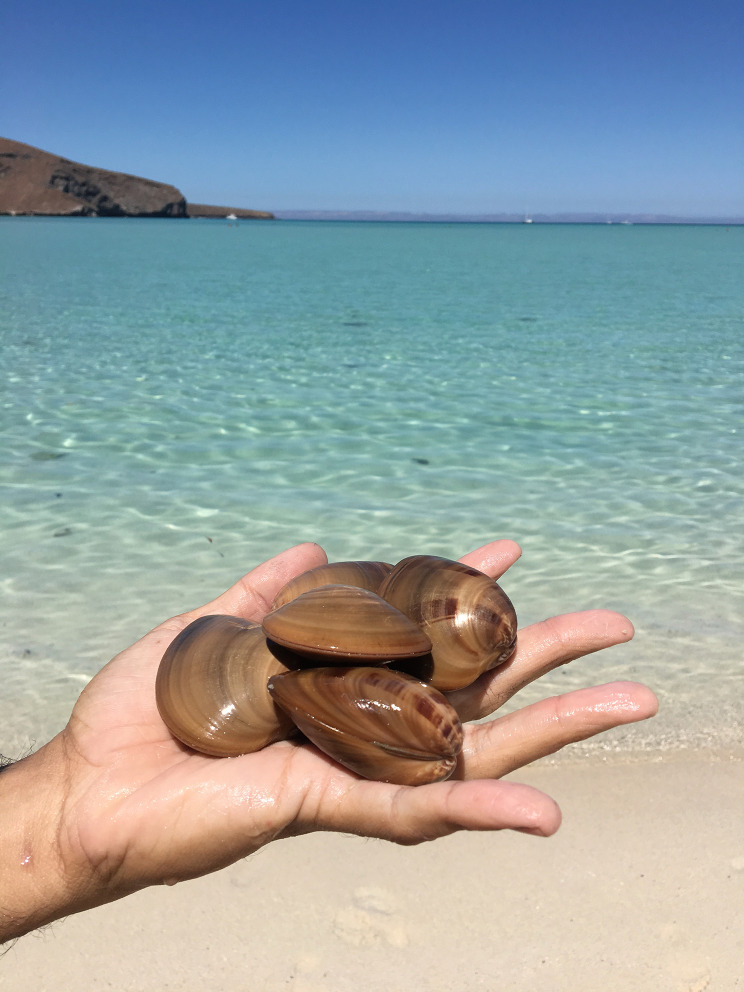
Mexican chocolate clams. Photo by K. E. Pellowe

The Town of Loreto, Baja California Sur, Mexico, lies along the sea between the *Sierra de la Giganta* Mountains and the Gulf of California (Fig. 1). Loreto is home to roughly 19 000 people, and the town’s economy depends on fisheries and tourism centered around the marine park it hosts (Instituto Nacional de Estadística y Geografía, INEGI 2017). Loreto Bay National Park (LBNP) is one of the largest marine protected areas in Mexico with an area of 2065 km^2^. The park contains varied marine and estuarine habitat types, including rocky reefs, seagrass beds, mangroves, and sandy habitats, and hosts a variety of permitted activities, including SCUBA diving, snorkeling, whale watching, wildlife viewing, kayaking, and commercial and sport fishing of select species (Comisión Nacional de Áreas Naturales Protegidas 2019). The waters of LBNP are home to 800 marine species, including the Mexican chocolate clam, *M. squalida* (Fig. 2). Mexican chocolate clams are soft-sediment burrowers that inhabit sandy-bottom habitat from the intertidal to depths of 160 m (Keen 1971). In Loreto Bay, Mexican chocolate clams are an important source of food and income for local fishing communities; they are among the top 5 species harvested by total biomass, and among the top 10 by total value (Pellowe and Leslie 2017).

Mexican chocolate clams are in demand year-round, sometimes despite seasonal harvest bans. The clams are a long-standing culinary tradition in the region, headline the menu of local restaurants, and are the focus of an annual gastronomic festival held on Loreto’s waterfront. The clam also serves as a symbol of community pride and connection to the sea; murals around Loreto Bay depict smiling clams reminding locals to fish responsibly. For many families in the region, Mexican chocolate clams provide supplementary food and income in times of limited resources, and serve as a safeguard against scarcity.

### Surveys

From February to May 2019, we carried out 48 surveys with residents of Loreto, Baja California Sur, Mexico to explore community perspectives on a range of ecosystem services. Prior to survey administration, questions were carefully reviewed, translated, and pretested with local volunteers to ensure the validity and clarity of questions in both English and Spanish (Groves et al. 2011). Surveys with less than 25% of questions completed (12 or fewer questions answered out of 48 total questions) were removed from the sample. Forty-two surveys were included in subsequent analyses. The participant population included adult community members (at least 18 years of age) of any occupation, residing in Loreto, Baja California Sur, Mexico at least 6 months of the year. Since Loreto has a large community of non-Mexican expat residents and Mexican nationals who are not originally from Loreto, the participant population included Mexican nationals originally from Loreto (Loretanos), other Mexican nationals who reside in Loreto, and nationals of other countries who reside in Loreto. Survey participants were recruited via purposive sampling of contacts established during previous fieldwork in the region. Sampling excluded residents with known economic dependence on the fishery (e.g., fishers), but included residents of Loreto thought to value Mexican chocolate clams based on their interest in our previous research. Participants were asked to answer survey questions from the perspective of their entire household, even if they themselves were not heads of household.

Due to variable literacy rates in the region, participants had the option of taking the survey themselves or having survey questions read aloud to them and their responses recorded by the researcher. Of 48 total surveys administered, 38 participants elected to take the survey themselves, and 10 elected to have the survey administered to them. Participants who took the survey themselves were less likely to complete it (32 of 38 surveys completed), as compared to participants who elected to have the survey administered to them by the researcher (10 out of 10 surveys completed). Informed consent was obtained from all participants prior to survey administration. Surveys were conducted in both Spanish and English. The survey instrument was written in both languages, allowing participants to read and respond in their preferred language. For surveys administered by the researcher, participants had the option to choose their preferred language for questions and responses. Each survey took approximately 10–20 min to complete. All procedures performed in this study were in accordance with the Ethical Standards of the Institutional Review Board (University of Maine IRB Permit # 2018-07-01).

Surveys were anonymous and collected information on the socioeconomic characteristics of households, how frequently members of their household harvest, buy, sell, and consume Mexican chocolate clams, and changes they have observed in the availability, market demand, quantity, quality, price, and size of clams over time (survey instrument available as Supplementary Material). Participants were then asked, using a three-item Likert scale (Likert 1932), to indicate whether they agreed, disagreed, or neither agreed nor disagreed with a set of statements (Table 1), each relating to an ecosystem service they and their household may receive from Mexican chocolate clams. Participants could also elect not to answer any questions of their choosing. Surveys were designed to elicit both use and non-use values. Selection of the services assessed in this study resulted from the compilation and adaptation of lists of multiple provisioning and cultural ecosystem services identified in diverse ecosystems (Rolston and Coufal 1991; Reed and Brown 2003; Millennium Ecosystem Assessment 2005; Raymond and Brown 2006). The final list of services consisted of values appropriate for assessment for individual species, and included general (household level), general (community level), life-sustaining (household level), life-sustaining (ecological), economic (household level), economic (community level), tourism, subsistence, scientific/learning, recreation, aesthetic, future use, historic, cultural, individual identity, community identity, existence, and intrinsic values (see Table 1 for full list of statements used to determine ecosystem service values).

**Table 1:** Value statements used to identify participants’ identification of ecosystem service values. Participants’ indication that they agreed with each statement (as opposed to having disagreed or said that they neither agreed nor disagreed) indicated their belief that Mexican chocolate clams provide the associated ecosystem service value. Intrinsic value was reverse-coded, where disagreement with the associated statement was taken as indication that the participant believed that Mexican chocolate clams have intrinsic value

Ecosystem service value assessed	Value statement
General	Chocolate clams are important to me and my family
	Chocolate clams are important to my community
Life sustaining	Chocolate clams help sustain me and my family
	Chocolate clams help sustain other animals in Loreto Bay
Economic	Chocolate clams provide income to my household
	Chocolate clams are important to the local economy
Tourism	Tourists spend money on chocolate clams when they visit Loreto
	Chocolate clams are a tourist attraction of Loreto
Subsistence	Chocolate clams provide some of my family’s basic needs
Scientific/learning	Chocolate clams are important for scientists to study
	Chocolate clams should be protected so that people can learn about them
Recreation	Chocolate clams are important for recreation, including exercise and fun
	It is fun or relaxing to look for or harvest chocolate clams
Aesthetic	Chocolate clams are beautiful
	Chocolate clams contribute to the unique beauty of Loreto
Future use	Chocolate clams should be conserved for future generations
	Chocolate clams should be conserved because I or my family might want to harvest them in the future
Historic	Chocolate clams are important because of their history in this area
Cultural	Chocolate clams are important to the culture of this area
Individual identity	Chocolate clams are an important part of who I am as an individual
Community identity	Chocolate clams are an important part of what it means to be a Loretano or to live in this area
Existence	Even when I don’t use chocolate clams, I like to know they are there
Intrinsic	Chocolate clams have value primarily because they provide benefits to people (reverse-coded)

We defined *general value at the household level* to be the overall importance of the clam to the participant’s household, while *general value at the community level* was the overall importance of the clam to the community. *Life-sustaining value at the household level* was considered to be the clams’ provision of life-sustaining benefits to the participant’s household, including food, income, or security, and *life-sustaining value at the ecological scale* was the clams’ role in sustaining other species or contributing to the broader coastal ecosystem. We defined *economic value* as the provision of income to the participant’s household, or to the broader community. *Tourism value* was defined as income generated from tourist activities (e.g., patronizing local restaurants to consume clams), or increased tourism as a result of the presence of Mexican chocolate clams in the region. *Subsistence value* was considered to be the provision of the participant’s basic needs, including food and/or income. *Scientific*/*learning value* was considered the potential for learning generated by the existence of the species, and the possibility for the advancement of science through studies of the species. *Recreational value* was defined as the potential for fun, relaxation, or enjoyment from harvesting or searching for clams. *Aesthetic value* was considered to be the beauty of the clam itself or its contribution to the overall beauty of the region. *Future use value* was defined as the ability of the participant or their household to harvest clams in the future, or the knowledge that future generations within the broader community would be able to harvest clams. *Historic value* was considered to be the importance of the clam to regional history, and *cultural value* as the contribution of the clam to regional culture and practice. We considered *individual identity value* to be the importance of the clam in constructing individual worldview and sense of self. *Community identity value* was considered to be the contribution of the clam to a shared sense of what it means to be a member of the Loreto community. *Existence value* was considered to be the satisfaction of knowing that the clam exists in Loreto Bay National Park, and *intrinsic value* was the belief that Mexican chocolate clams have inherent value, outside of human interaction.

The ecosystem services of tourism, scientific/learning, recreation, and aesthetic values were assessed each with two survey questions, and an average was taken from the two responses to determine whether participants identified these values from Mexican chocolate clams. Additionally, we assessed the following values both at the individual and the community level through two separate questions: general, economic, future use, and identity. For open-ended survey questions, including questions on the nature of changes observed, and participants’ perspectives on why changes had occurred, responses were coded into categories. These categories emerged from analysis of participant responses by the researcher who conducted the surveys. Responses that were cited by two or more participants were considered response categories.

We analyzed separately the responses of three participant groups: Mexican nationals originally from Loreto; Mexican nationals not originally from Loreto; and foreign nationals. Maps and figures were created using R statistical software (R Core Team 2020) and the R packages ggplot2, ggspatial, rnaturalearth, and wesanderson (Wickham 2016; South 2017; Karthik and Wickham 2018; Dunnington 2020).

## Results

### Participant demographics and use behavior

Of 42 survey participants whose responses were included in the final analyses, 52% were Mexican nationals (of which, 40% were originally from Loreto) and 48% were nationals of other countries, including the United States, Canada, Germany, Australia, Chile, Switzerland, and the United Kingdom. These numbers also correspond to the number of surveys conducted in Spanish (52%) and English (48%). Overall, 19% of survey participants were Mexican nationals originally from Loreto or Loretanos. Participants varied in the average length of time they had lived in Loreto, their mean monthly household income, mean household size, and reported frequency of use of Mexican chocolate clams (Table 2).

**Table 2:** Demographic characteristics and reported use behavior by participant group

Demographic characteristics and use behavior	Participant group		
	Mexican nationals from Loreto	Mexican nationals from elsewhere	Foreign nationals
*n*	8	14	20
Mean time in Loreto (years)	42	17	8
Mean monthly household income (US Dollars)	654	917	3924
Mean household size (number of people)	3.9	2.4	2.0
Reported harvest of clams (times per year)	4.0	14.5	0.4
Reported purchase of clams (times per year)	10.9	19.7	17.7
Reported sales of chocolate clams (times per year)	7.4	0.0	0.0
Reported consumption of chocolate clams (times per year)	7.6	20.8	19.1

None of the participants of any group reported clamming as a source of household income, despite Loretano participants reporting selling chocolate clams 7.4 times per year on average. 67% of Loretano participants responded that they had harvested Mexican chocolate clams at some point in the past. 50% of other Mexican participants and 25% of foreign participants reported harvesting Mexican chocolate clams at some point in the past. The participants originally from Loreto who indicated that they regularly harvest or used to regularly harvest Mexican chocolate clams had 13.3 years of harvest experience, on average, with a range of 5 to 20 years of experience. Loretano participants also had, on average, 34.6 years of experience buying Mexican chocolate clams, with a range of experience from 1 to 82 years. Mexican participants not originally from Loreto reported an average of 4.1 years of harvest experience and 15.7 years of buying experience, while foreign participants reported 8.7 years of harvest experience and 8.4 years of buying experience, on average.

### Perceptions of change

**Fig. 3:**
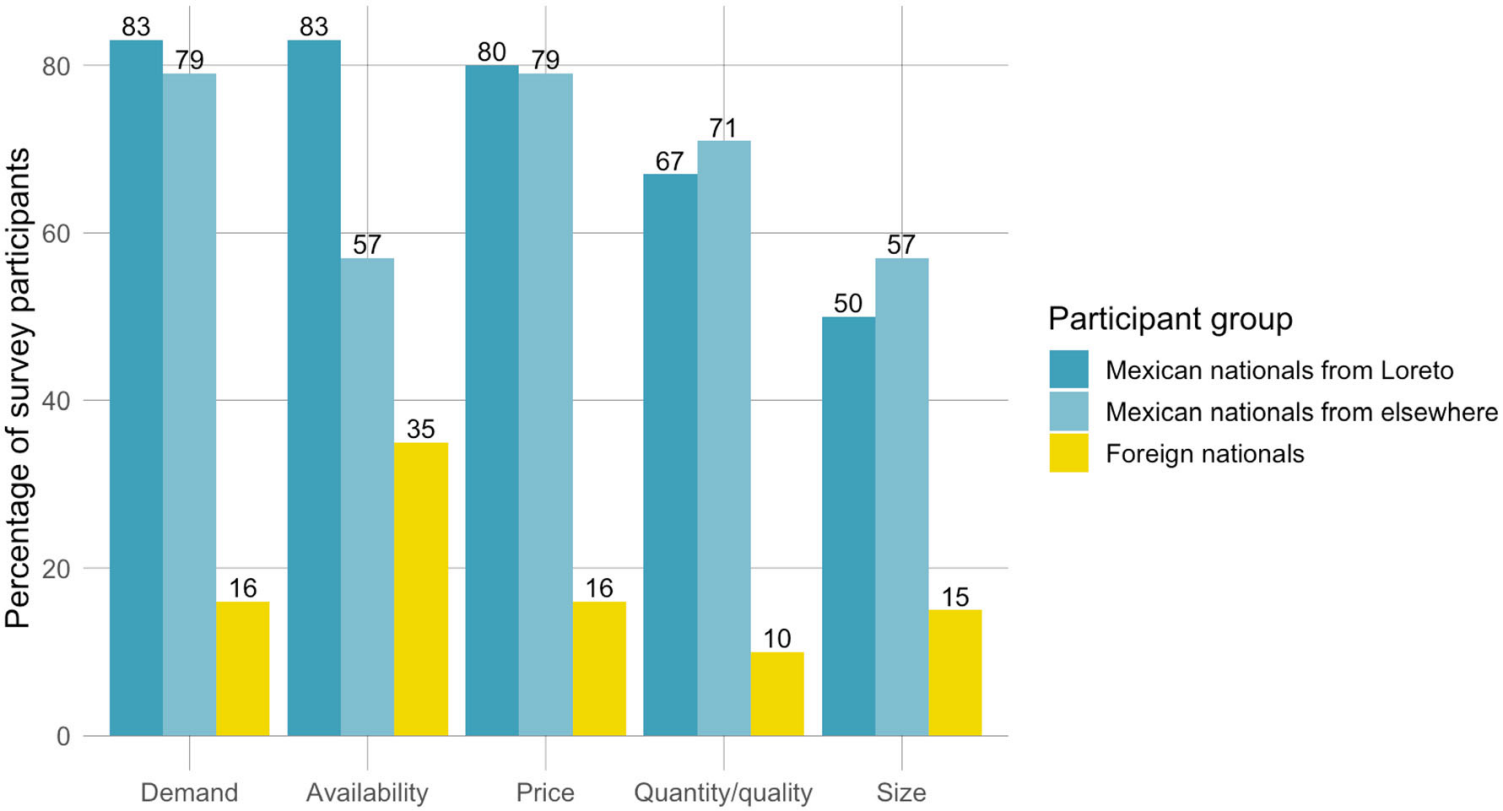
Survey participants have observed changes in Mexican chocolate clams, including in market demand, availability, price, quantity and/or quality, and size of individual clams. Percentages of survey participants who have observed these changes vary by type of change and participant group. Participant groups include Mexican nationals originally from Loreto (*n* = 8), Mexican nationals not originally from Loreto (*n* = 14), and foreign nationals (*n* = 20). The most highly cited changes were in market demand and availability of clams, followed by price, quantity and/or quality, and size of clams

**Fig. 4:**
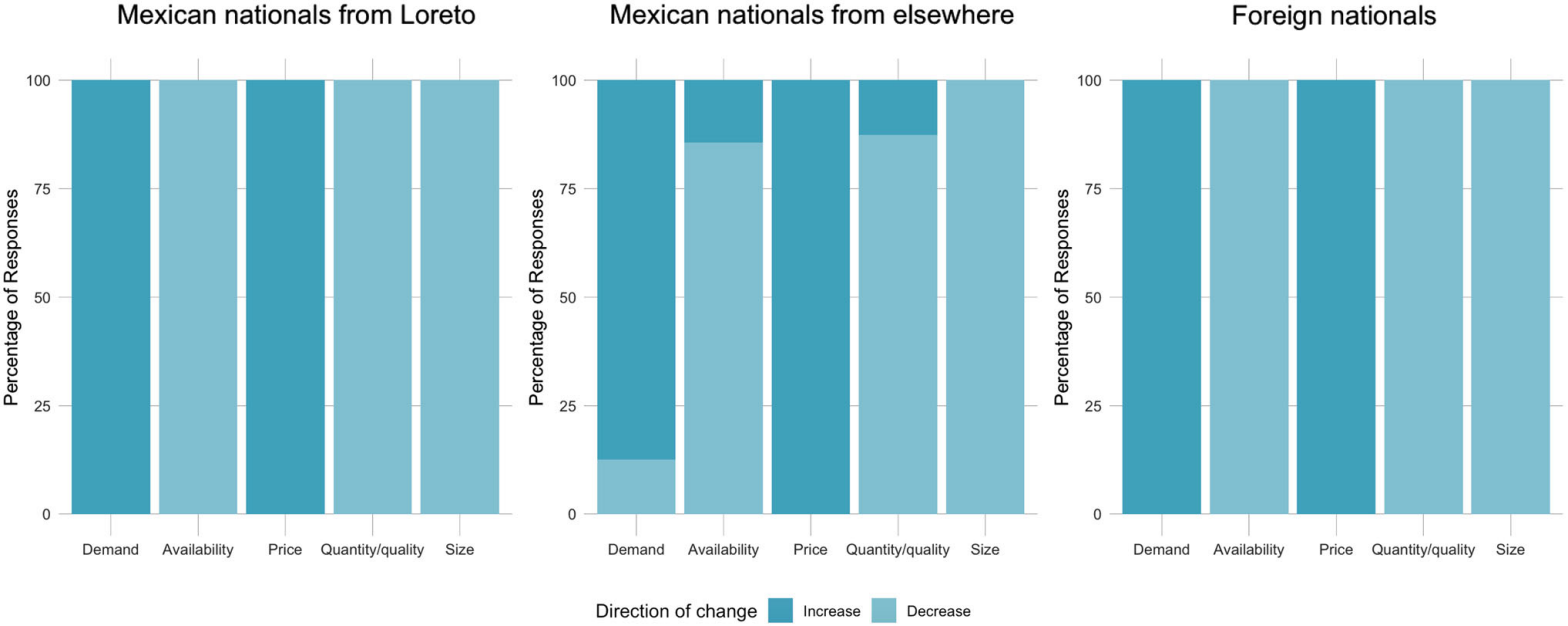
Survey participants who provided qualitative descriptions of changes observed in Mexican chocolate clams largely agreed on the directionality of change. Participants who provided information on the nature of changes observed included Mexican nationals originally from Loreto (*n* = 8), Mexican nationals not originally from Loreto (*n* = 14), and foreign nationals (*n* = 20)

83% of Loretanos surveyed, 93% of Mexican participants not originally from Loreto, and 50% of foreign participants said they had noticed at least one change over time in terms of market demand, quantity, quality, size, price, and/or availability of the species. Observations of change varied by participant group and type of change (Fig. 3). Differing levels of observations of change may have been due, in part, to varying lengths of time spent in the region among the three participant groups. Participants largely agreed on the directionality of changes (Fig. 4), and reported that demand for and price of clams had increased over time, while the availability, quantity and/or quality, and average size had decreased.

Despite the fact that most participants had noticed qualitative changes related to the market demand, quantity, quality, size, price, and/or availability of Mexican chocolate clams, none of the participants in any group said that the changes they had observed had directly affected their household. When asked whether they had any thoughts on why these changes had occurred, participant responses fell into four main categories, in the order of most to least cited: fisheries management, overfishing, increased demand, and environmental change (Table 3).

**Table 3:** Participant perspectives on why changes that have occurred fell into four primary categories, in order of most to least cited: fisheries management, overfishing, increased demand, and environmental change

Do you have any thoughts on why these changes have occurred?		
Response category	Times cited	Illustrative quote(s)
Fisheries management	9	“It’s because of poor management of the clam”, “It’s because of the cooperatives that use a compressor to harvest”
Overfishing	9	“The uncontrolled exploitation”
Increased demand	4	“It’s a tourist town, and this is the dish that represents our town”; “There is more consumption now”; “Supply and demand- there are more people in Loreto now”
Environmental change	3	“The temperature—sometimes it’s too warm”

### Ecosystem service values

All but one ecosystem service value assessed was reported to be provided by chocolate clams to survey participants: this was personal economic value (assessed with the statement, “Chocolate clams provide income to my household”). This is consistent with the lack of reported income from clamming among those surveyed. Relatedly, none of the participants originally from Loreto, 7% of participants from elsewhere in Mexico, and none of the foreign participants reported that their household receives life-sustaining value from Mexican chocolate clams (assessed with the statement, “Chocolate clams help sustain me and my family”), and 33% of Loretano participants, 14% of Mexican participants not originally from Loreto, and none of the foreign participants reported receiving subsistence value (assessed with the statement, “Chocolate clams provide some of my family’s basic needs”). However, several participants noted that while their household does not receive life-sustaining or subsistence value from Mexican chocolate clams, many other households in the community do. In fact, all participants originally from Loreto, all Mexican participants not originally from Loreto, and 90% of foreign participants agreed that Mexican chocolate clams are important to the community of Loreto (assessed with the statement, “Chocolate clams are important to my community”). Participants also agreed that chocolate clams help to shape the community identity of Loreto; 100% of Loretano participants, 79% of Mexican participants not originally from Loreto, and 60% of foreign participants agreed with the statement, “Chocolate clams are an important part of what it means to be a Loretano or to live in this area.” Perhaps unsurprisingly, more participants originally from Loreto than participants from elsewhere felt that the clam also played a role in shaping their individual identity; 50% of Loretano participants agreed with the statement, “Chocolate clams are an important part of who I am as an individual,” as compared to 7% of Mexican participants not originally from Loreto, and none of foreign participants.

**Fig. 5:**
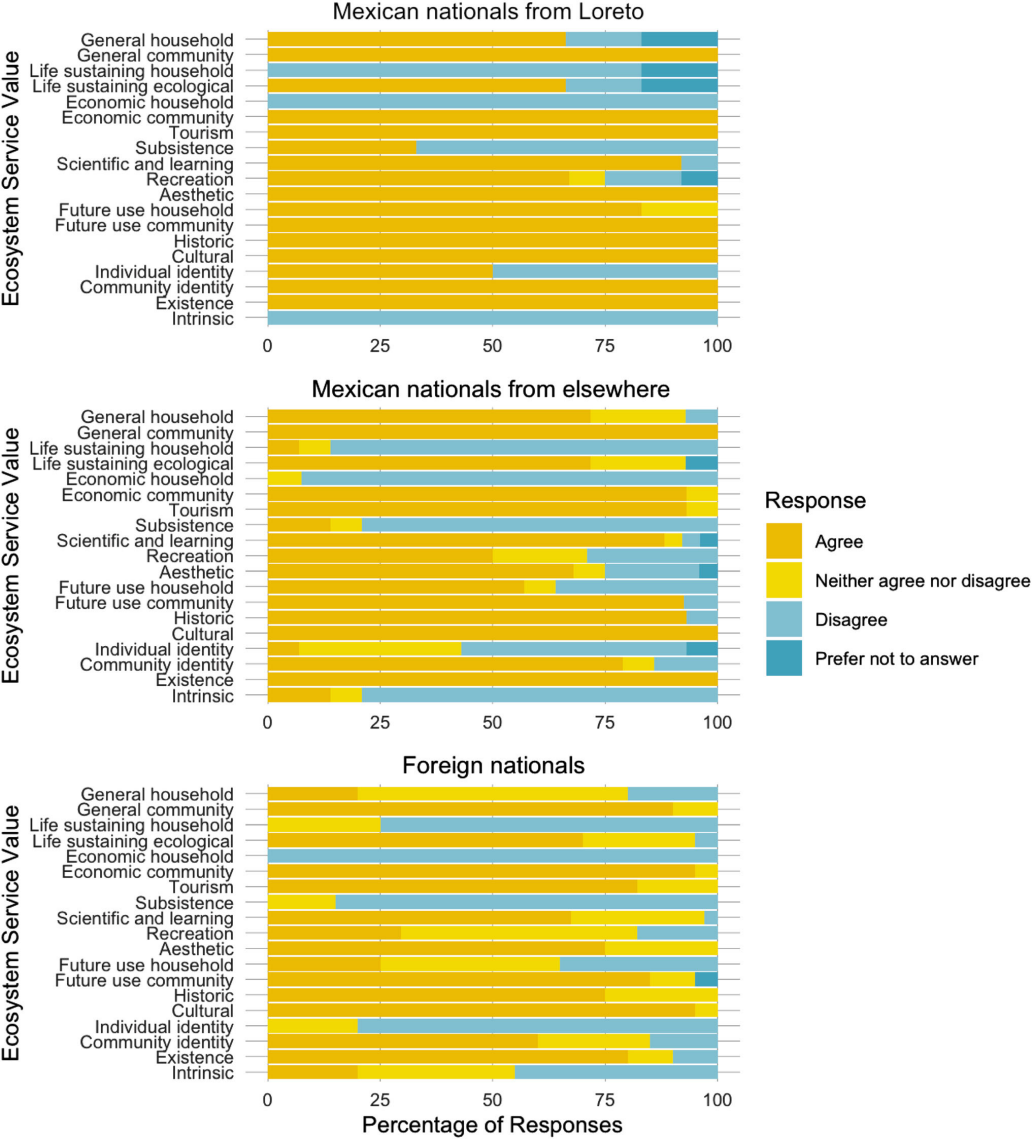
Ecosystem services with the highest levels of agreement among participants across participant groups include general community value, economic community value, cultural value, and tourism value. Ecosystem services with the lowest levels of agreement among participants across groups include life-sustaining household, economic household, and subsistence values. For values with two corresponding statements in surveys (tourism, scientific and learning, recreation, and aesthetic values), response percentage represents the average response for the two statements. For intrinsic value, which was reverse-coded, responses have been reversed for ease of comparison with other values. Participant groups include Mexican nationals originally from Loreto (*n* = 8), Mexican nationals from elsewhere (*n* = 14), and foreign nationals (*n* = 20)

While participants surveyed reported that their households do not receive economic value from Mexican chocolate clams (0% agreement with the statement, “Chocolate clams provide income to my household” across all three participant groups), nearly all agreed that the clams provide economic value to the community (100% of Loretano participants, 86% of Mexican participants not originally from Loreto, and 100% of foreign participants agreed with the statement, “Chocolate clams are important to the local economy”). Additionally, nearly all participants agreed that the clam contributes to local tourism (100% Loretano, 93% other Mexican participants, and 83% foreign participant agreement with the two statements, “Tourists spend money on chocolate clams when they visit Loreto” and “Chocolate clams are a tourist attraction of Loreto”). Additional ecosystem services with high levels of agreement among survey participants include cultural value (100%, 100%, and 95% agreement among the three groups, respectively, with the statement, “Chocolate clams are important to the culture of this area”), historic value in the region (100%, 93%, and 75% agreement among the three groups, respectively, with the statement, “Chocolate clams are important because of their history in this area”), existence value (100%, 100%, and 80% agreement among the three groups, respectively, agreement with the statement “Even when I don’t use chocolate clams, I like to know they are there”), and future community use value (100%, 86%, and 85% agreement among the three groups, respectively, agreement with the statement, “Chocolate clams should be conserved for future generations”). A full report of values assessed and responses for each participant group can be found in Fig. 5.

## Discussion

Mexican chocolate clams provide a host of ecosystem services to households in the Loreto region that include both provisioning and cultural services. As bivalves, the clams also provide regulating services in the form of water filtration (Millennium Ecosystem Assessment 2005). The multitude of ecosystem services provided by the clams are not explicitly recognized in fisheries management; current management focuses on ecological and economic factors. We find that in addition to the provisioning services produced by the fishery, households in the Loreto region derive many cultural ecosystem services from Mexican chocolate clams. Community members agree that in addition to economic value generated by the Mexican chocolate clam fishery, this species also contributes to tourism, scientific/learning, recreation, aesthetic, historic, cultural, community identity, and existence values. This finding is consistent with other ecosystem service valuation studies that have found that high percentages of local stakeholders recognize their local ecosystems’ capacity to produce diverse ecosystem services including social and cultural values (Martín-López et al. 2012; Oteros-Rozas et al. 2014). Community members report receiving many types of ecosystem services from the species, which supports our hypothesis that Mexican chocolate clams provide a diversity of both provisioning and cultural values to the community of Loreto. None of the participants in the survey reported relying on income from clam harvest, yet nearly half of all participants indicated that they have collected Mexican chocolate clams at some point in the past, and a third said that they collect clams at least once per year. This indicates that residents of Loreto who are not fishers also participate in the harvest of Mexican chocolate clams, and that the fishery itself is much more heterogeneous than accounted for by current fisheries management. This finding is supported by the previous work demonstrating that multiple fisher types harvest Mexican chocolate clams in Loreto, and that marginalized fisher groups are excluded from fisheries management processes (Pellowe and Leslie 2019).

Fisheries management decisions have consequences not only for fishers directly engaged in resource extraction, but also for the broader coastal community. In communities like Loreto, where relatively few individuals engage in regular harvest of the Mexican chocolate clam as commercial fishers (Pellowe and Leslie 2019), the values provided by the species to the broader coastal community are diverse and significant. Accounting for diverse ecosystem services and community perspectives in management requires first, identifying the values and aims of the community, and then, creating management that accounts for trade-offs and conflicts among multiple priorities (Loomis and Paterson 2014). Fisheries management in Baja California Sur is improving in its ability to integrate the heterogeneity of ecological systems into policies, but the sociocultural richness of fisheries systems and coastal communities remains largely unaccounted for (see for example, Finkbeiner and Basurto 2015; Leslie et al. 2015). An ecosystem service assessment like what we present here can help inform ecosystem-based management that better incorporates sociocultural richness (Rosenberg and McLeod 2005).

Cultural ecosystem services underpin stakeholders’ values and preferences (Russell et al. 2013). However, translating ecosystem service assessments into policy has many challenges, including reconciling the legitimacy of diverse knowledge types, and finding pathways to turn such knowledge into action (Posner et al. 2016). The purposive inclusion of cultural ecosystem services in these broader assessments is one way to ensure that the sociocultural richness of human–nature interactions as well as the knowledge and values of diverse stakeholders are incorporated into management (Chan et al. 2012a; Loomis and Paterson 2014; Scholte et al. 2015). Previous work assessing diverse ecosystem services for management has largely focused on terrestrial environments (e.g., Martín-López et al. 2012; Oteros-Rozas et al. 2014; Dickinson and Hobbs 2017), but there is growing interest in the utility of such approaches for integrating diverse values into the management of marine systems (Rees et al. 2010; Klain and Chan 2012; Loomis and Paterson 2014; Gregr et al. 2020).

Stakeholders’ perceptions of change also provide valuable information about changes in the delivery of benefits that can help to identify management priorities (Martín-López et al. 2012, 2013; Oteros-Rozas et al. 2014). In this study, perceptions of change provide important insight into how community members may make decisions regarding the clams and resulting marine conservation outcomes, since stakeholder perceptions of ecological conditions underpin environmental behavior (Gobster et al. 2007). Stakeholders’ perceptions of change have been important to understand temporal shifts in other marine populations and ecosystems in the Gulf of California (Sala et al. 2004; Sáenz-Arroyo et al. 2005a, b, 2006). A study of fisher perceptions of trends in the abundance of the Gulf grouper (*Mycteroperca jordani*) revealed dramatic declines in abundance that occurred prior to the collection of fisheries data in the Gulf of California, and were thus unaccounted for in fisheries management (Sáenz-Arroyo et al. 2005b). Alongside fisheries statistics and surveys, fishers’ observations of change over time have also revealed shifts in the species composition of coastal ecosystems of the Gulf of California, from mostly large, long-lived species in higher trophic levels to mostly small, short-lived species in lower trophic levels (Sala et al. 2004). Perceptions of change in marine environments are particularly valuable where long-term monitoring data are not available, as they contribute critical information for setting appropriate management targets (Sala et al. 2004).

Participants in this study reported changes in Mexican chocolate clams over time in the form of increased market demand, higher prices, reduced availability, reduced quantity and quality, and smaller size. Changes were reported at higher rates by Mexican nationals than foreign nationals surveyed, perhaps because the Mexican nationals surveyed had lived in Loreto longer and had more years of experience harvesting and buying clams. Observed changes in demand, price, availability, quantity, quality, and size of clams affect the delivery of ecosystem services and reveal potential priorities for management. Survey participants proposed several possible causes of observed changes including fisheries management, overfishing, increased demand for chocolate clams, and environmental change. Although survey participants were predominantly non-fishers, the nature of their observations of change and attributed causes of change echo those reported by harvesters of the Mexican chocolate clam in previous studies (Pellowe and Leslie 2019). Harvesters reported declines in Mexican chocolate clam populations over time, which they attributed to increased fishing effort resulting from changes in fisheries management (Pellowe and Leslie 2019). Stakeholders’ observations of change provide information on potential shifts in clam populations and the ecosystem services they generate that is critical for effective design and implementation of management strategies. Such studies are particularly important in data-limited fisheries, like the Mexican chocolate clam fishery (Pellowe and Leslie 2020), where long-term abundance data may not be available.

While survey participants did not feel acutely impacted by the changes they had observed, they believed other households in their community were affected. Similarly, community members we surveyed acknowledged the importance of the services provided by Mexican chocolate clams to the broader community of Loreto, even if they themselves did not feel that they received every service. Survey participants were more likely to report the delivery of both provisioning and cultural ecosystem services at the community level, especially for the values of general importance, life-sustaining value, economic value, future use value, and identity value, than they were to report the delivery of the same services at the individual or household level. Community members in Loreto recognize the community value of the Mexican chocolate clam and the impacts of change on the delivery of ecosystem services at the community level.

Of the values assessed in this study, the most important ecosystem services that the Mexican chocolate clam provides to the community of Loreto include economic, tourism, future use, cultural, and existence values. Many locals recall childhood memories of collecting Mexican chocolate clams during family trips to the beach, learning to dig for clams in the sand with their toes, or holding their breath to grab a clam from the ocean floor (Pellowe unpublished data). Survey participants originally from Loreto were more likely to agree that the clam contributes to their individual identity than participants from elsewhere. However, most participants surveyed, regardless of their place of origin, agreed that the clam is an important part of what it mean to be a member of the Loreto community.

Considering the wide recognition of cultural ecosystem services provided to Loreto households, and the clam’s contribution to local identity, the Mexican chocolate clam may be considered a cultural keystone species. Cultural keystone species are “culturally salient species that shape in a major way the cultural identity of a people” (Garibaldi and Turner 2004, p. 4). Such species are defined by the key role they play in defining cultural identity and are characterized by their high cultural significance. Cultural keystone species are also marked by their provision of important ecosystem services, particularly cultural value (Butler et al. 2012). The concept of the cultural keystone species highlights the importance of communities’ relationship to place, and the conservation status of these species may be a starting point for identifying management priorities (Garibaldi and Turner 2004). In the Torres Strait Islands in Australia, two cultural keystone species, turtles and dugongs, were catalysts for a shift towards adaptive co-management, which involves the formal sharing of power between local stakeholders and regional fisheries managers, and the formal integration of local ecological knowledge into resource governance (Butler et al. 2012). Their findings highlight the value of cultural keystone species as catalysts for the integration of local knowledge into marine resource governance to enhance fisheries policy and protect the future delivery of ecosystem services (Butler et al. 2012).

In Loreto, embracing Mexican chocolate clams as a cultural keystone species may facilitate greater community participation in marine resource management decisions that is reflective of the heterogeneity among those involved in the fishery, both directly and indirectly. It may also result in the integration of local ecological knowledge into future policy decisions including accounting for community and fisher observations of change and investigating possible causes of change in order to identify management priorities. Managing for Mexican chocolate clams' diverse values might include protecting habitat, regulating water quality, and privileging low-impact fishing practices to safeguard the future delivery of both provisioning and cultural ecosystem services. These practices would serve not only to conserve Mexican chocolate clams and the benefits they provide to Loreto households, but would also benefit many other marine species in Loreto’s nearshore waters including fish, rays, octopus, and other molluscs.

While this study provides important insights about community members’ perceptions of change in the clam fishery and the provisioning and cultural ecosystem services that Mexican clams provide to households in the Loreto region, a larger sample size of survey participants would be needed to generalize our findings to the broader population of Loreto residents. A future study with a larger number of participants, systematically recruited to ensure representativeness of the socioeconomic makeup of Loreto households, could confirm whether our findings apply more broadly to the general population. Our participant pool consisted of many households of middle and upper socioeconomic status owing to the fact that nearly half of the participants surveyed were expats and nationals of the United States, Canada, and the European Union. The skewed socioeconomic characteristic of the participant pool in this study is a result of the purposive sampling method used to recruit participants. Future work should include surveys conducted with a more representative and wider participant pool in order to verify whether our findings hold true for Loreto residents more broadly. To investigate further the clam’s role in shaping community identity in Loreto as a cultural keystone species, the inclusion of more participants originally from Loreto should be prioritized in future work. The participants in this study did not rely on clams as a source of income, sustenance, or other basic needs, and we anticipate that the inclusion of more low-income households would lead to higher reporting of these services. Additionally, future work should include an expansion of the range of responses to value statements, in order to facilitate comparisons in the strength of participant response to different values. The three-item Likert scale employed in this study to assess survey participants’ agreement or disagreement with ecosystem service value statements could be expanded to a Likert scale that includes a greater range of degrees of agreement and disagreement. This would produce a richer understanding of participants’ experience of diverse values, as well as the relative importance of provisioning and cultural ecosystem services.

The social and cultural values of species and ecosystems shape human–nature interactions, yet are often overlooked in decision-making and design of marine management (Chan et al. 2011). If such values are not explicitly understood and accounted for, they are likely to be poorly represented in natural resource policy (Klain and Chan 2012). Assessing these values and incorporating them into management creates robust policies that protect the future provision of valuable ecosystem services. Managing for a narrow set of ecosystem services may not only ignore other important values that a species or ecosystem provides to human communities, but can also reduce the fishery’s capacity to cope with future disturbance (Gordon et al. 2008; Bennett et al. 2009). Understanding the full suite of ecosystem services provided by fished species is a critical step in designing resource management that protects crucial benefits, while considering trade-offs among the diverse values and priorities of coastal communities.

## Electronic supplementary material

Below is the link to the electronic supplementary material.

Supplementary file1 (PDF 109 kb)
