# Supplementary material for: Ecosystem service lens reveals diverse community values of small-scale fisheries
Source: Ambio. 2020 Nov 3;50(3):586–600. doi: 10.1007/s13280-020-01405-w (PMC7882666; doi:10.1007/s13280-020-01405-w)
Supplement: Supplementary file 1 — Supplementary file1 (PDF 109 kb) [file 13280_2020_1405_MOESM1_ESM.pdf]

Ambio

Electronic Supplementary Material
This supplementary material has not been peer reviewed

Title: **Ecosystem service lens reveals diverse community values of small-scale fisheries**

Kara E. Pellowe and Heather M. Leslie

Supplementary Materials. Survey instrument used to assess community values of Mexican chocolate clams in Loreto, B.C.S., Mexico

Survey No. / *Nu. de encuesta* ____ Date / *Fecha*_________

Do you have any questions about the study or the consent form before beginning?
¿Tiene algunas preguntas sobre el estudio o el documento de consentimiento antes de empezar?

Household socioeconomic characteristics / *Características socioeconómicas del hogar*

1. Location of household (town, neighborhood) / *Ubicación de hogar (pueblo o barrio)*

____

2. Size of household / *Cuántos habitantes hay en su hogar*

____

3. Ages of household members / *Edades de miembros de hogar*

____

1. Highest school grade level or degree achieved by household adults / *El grado de escuela o licenciatura más alta alcanzada de los adultos de hogar*

____

2. Employment status of household adults / *Estado de empleo de los adultos de hogar*

    Employed full-time / *Trabajo de tiempo completo*

    Employed part-time / *Trabajo temporal*

    Unemployed / *Desempleados*

3. Birthplaces of household adults / *Lugares de nacimiento de los adultos de hogar*

____

    a. How many years have you lived in this region? *Cuántos años ha vivido en esta región?*

____

4. Where did your household income come from in the past year? *¿De dónde vinieron sus ingresos de casa en el año pasado?*

Type of income<br>*Tipo de ingresos*	Employment<br>*Tipo de empleo*	Amount per month<br>*Ingreso por mes*	Frequency<br>(months/year)<br>*Frecuencia*<br>*(meses por año)*
Primary income<br>*Empleo principal*			
Additional 1<br>*Adicional 1*			
Additional 2<br>*Adicional 2*			
Additional 3<br>*Adicional 3*			

Current clam use / *Uso presente de almejas*

Answer for your entire household / *Conteste para toda su casa*

1. How frequently does a member of your household collect/harvest chocolate clams? *¿Con qué frecuencia colectan o sacan almejas chocolatas?*

    ____x per year / *por año*
    ____ x per month / *por mes*
    ____x per week / *por semana*

2. How frequently does your household buy chocolate clams? *¿Con qué frequencia compran almejas chocolatas?*

    ____x per year / *por año*
    ____ x per month / *por mes*
    ____x per week / *por semana*

3. How frequently does your household sell chocolate clams? *¿Con qué frecuencia venden almejas chocolatas?*

    ____x per year / *por año*
    ____ x per month / *por mes*
    ____x per week / *por semana*

4. How frequently does your household eat chocolate clams? *¿Con qué frecuencia comen almejas chocolatas?*

    ____x per year / *por año*
    ____ x per month / *por mes*
    ____x per week / *por semana*

Historic clam use / *Uso histórico de almejas*

1. Have you ever collected chocolate clams for any purpose? *¿Ha sacado almejas chocolatas por qualquier razón?*

   ____

2. If you collect or harvest chocolate clams, how long have you been collecting them? *Si ha sacado almejas chocolatas, ¿cuántos años las ha estado sacando o las ha sacado?*

   ____

3. If you buy chocolate clams, how long have you been buying them? *Si compra almejas chocolatas, ¿por cuántos años las ha comprado?*

   ____

4. Have you noticed any changes over time in the market or demand for clams? *¿Ha notado algún cambio en el mercado o la demanda para almejas chocolatas?*

   ____

   a. Have you noticed any changes over time in the quantity or quality of clams? *¿Ha notado algún cambio en la cantidad disponible o la calidad de almejas?*

   ____

   b. Have you noticed any changes over time in the size of clams? *¿Ha notado algún cambio en el tamaño de almejas?*

   ____

   c. Have you noticed any changes over time in the price of clams? *¿Ha notado algún cambio en el precio de almejas?*

   ____

   d. Have you noticed any changes in the availability of clams? *¿Ha notado algún cambio en la disponibilidad de almejas?*

   ____

5. Do have any thoughts on why these changes have occurred? *¿Tiene alguna idea en por qué han ocurrido estos cambios?*

   ____

6. Have these changes affected you and your household? *¿Han afectado estos cambios a su hogar?*

   ____

Chocolate Clam Values / *Valores de almejas chocolatas*

The following set of questions will ask about the values chocolate clams provide to your household. Please indicate whether you agree or disagree with each statement.

Se le preguntara sobre los valores que proporcionan almejas chocolatas a su hogar. Por favor indique si está de acuerdo o en desacuerdo con cada declaración.

7. Chocolate clams are important to me and my family. *A mi y a mi familia nos importan las almejas chocolatas.*

   __Agree / *De acuerdo*
   __Neither agree nor disagree / *Ni de acuerdo ni en desacuerdo*
   __Disagree / *En desacuerdo*
   __Prefer not to answer / *Prefiero no responder*

8. Chocolate clams are important to my community. *A mi comunidad le importan las almejas chocolatas.*

   __Agree / *De acuerdo*
   __Neither agree nor disagree / *Ni de acuerdo ni en desacuerdo*
   __Disagree / *En desacuerdo*
   __Prefer not to answer / *Prefiero no responder*

9. Chocolate clams help sustain me and my family. *Las almejas chocolatas ayudan a sostener a mí y a mi familia.*

   __Agree / *De acuerdo*
   __Neither agree nor disagree / *Ni de acuerdo ni en desacuerdo*
   __Disagree / *En desacuerdo*
   __Prefer not to answer / *Prefiero no responder*

10. Chocolate clams help sustain other animals in Loreto Bay. *Las almejas chocolatas ayudan a sostener otros animales en la Bahía Loreto.*

   __Agree / *De acuerdo*
   __Neither agree nor disagree / *Ni de acuerdo ni en desacuerdo*
   __Disagree / *En desacuerdo*
   __Prefer not to answer / *Prefiero no responder*

11. Chocolate clams provide income to my household. *Las almejas chocolatas proporcionan ingresos a mi hogar.*

__Agree / *De acuerdo*
__Neither agree nor disagree / *Ni de acuerdo ni en desacuerdo*
__Disagree / *En desacuerdo*
__Prefer not to answer / *Prefiero no responder*

12. Chocolate clams are important to the local economy. *Las almejas chocolatas son importantes a la economía local.*

__Agree / *De acuerdo*
__Neither agree nor disagree / *Ni de acuerdo ni en desacuerdo*
__Disagree / *En desacuerdo*
__Prefer not to answer / *Prefiero no responder*

13. Tourists spend money on chocolate clams when they visit Loreto. *Los turistas gastan dinero en almejas chocolatas cuando visitan Loreto.*

__Agree / *De acuerdo*
__Neither agree nor disagree / *Ni de acuerdo ni en desacuerdo*
__Disagree / *En desacuerdo*
__Prefer not to answer / *Prefiero no responder*

14. Chocolate clams are a tourist attraction of Loreto. *Las almejas chocolatas son una atracción turística en Loreto.*

__Agree / *De acuerdo*
__Disagree / *En desacuerdo*
__Neither agree nor disagree / *Ni de acuerdo ni en desacuerdo*
__Prefer not to answer / *Prefiero no responder*

15. Chocolate clams provide some of my family's basic needs. *Las almejas chocolatas proporcionan algunas de las necesidades básicas de mi familia.*

__Agree / *De acuerdo*
__Neither agree nor disagree / *Ni de acuerdo ni en desacuerdo*
__Disagree / *En desacuerdo*
__Prefer not to answer / *Prefiero no responder*

16. Chocolate clams are important for scientists to study. *Las almejas chocolatas son importantes para que los científicos las estudien.*

__Agree / *De acuerdo*
__Neither agree nor disagree / *Ni de acuerdo ni en desacuerdo*
__Disagree / *En desacuerdo*
__Prefer not to answer / *Prefiero no responder*

17. Chocolate clams should be protected so that people can learn about them. *Las almejas chocolatas deben ser protegidas para que la gente puede aprender sobre ellas.*

__Agree / *De acuerdo*
__Neither agree nor disagree / *Ni de acuerdo ni en desacuerdo*
__Disagree / *En desacuerdo*
__Prefer not to answer / *Prefiero no responder*

18. Chocolate clams are important for recreation, including exercise and fun. *Las almejas chocolatas son importantes para la recreación en cuanto a ejercicio y diversión.*

__Agree / *De acuerdo*
__Neither agree nor disagree / *Ni de acuerdo ni en desacuerdo*
__Disagree / *En desacuerdo*
__Prefer not to answer / *Prefiero no responder*

19. It is fun or relaxing to look for or harvest chocolate clams. *Es divertido o relajante buscar o sacar las almejas chocolatas.*

__Agree / *De acuerdo*
__Neither agree nor disagree / *Ni de acuerdo ni en desacuerdo*
__Disagree / *En desacuerdo*
__Prefer not to answer / *Prefiero no responder*

20. Chocolate clams are beautiful. *Las almejas chocolatas son bellas.*

__Agree / *De acuerdo*
__Neither agree nor disagree / *Ni de acuerdo ni en desacuerdo*
__Disagree / *En desacuerdo*
__Prefer not to answer / *Prefiero no responder*

21. Chocolate clams contribute to the unique beauty of Loreto. *Las almejas chocolatas contribuyen a la belleza única de Loreto.*

__Agree / *De acuerdo*
__Neither agree nor disagree / *Ni de acuerdo ni en desacuerdo*
__Disagree / *En desacuerdo*
__Prefer not to answer / *Prefiero no responder*

22. Chocolate clams should be conserved for future generations. *Almejas chocolatas deben ser conservadas para futuras generaciones.*

__Agree / *De acuerdo*
__Neither agree nor disagree / *Ni de acuerdo ni en desacuerdo*
__Disagree / *En desacuerdo*
__Prefer not to answer / *Prefiero no responder*

23. Chocolate clams should be conserved because I or my family might want to harvest them in the future. *Almejas chocolatas deben ser conservadas porque yo o mi familia podría querer sacarlas en el futuro.*

__Agree / *De acuerdo*
__Neither agree nor disagree / *Ni de acuerdo ni en desacuerdo*
__Disagree / *En desacuerdo*
__Prefer not to answer / *Prefiero no responder*

24. Chocolate clams are important because of their history in this area. *Las almejas chocolatas son importantes para su historia en esta area.*

__Agree / *De acuerdo*
__Neither agree nor disagree / *Ni de acuerdo ni en desacuerdo*
__Disagree / *En desacuerdo*
__Prefer not to answer / *Prefiero no responder*

25. Chocolate clams are important to the culture of this area. *Las almejas chocolatas son importantes a la cultura de esta area.*

__Agree / *De acuerdo*
__Neither agree nor disagree / *Ni de acuerdo ni en desacuerdo*
__Disagree / *En desacuerdo*
__Prefer not to answer / *Prefiero no responder*

26. Chocolate clams are an important part of who I am as an individual. *Las almejas chocolatas son una parte importante de quien soy como individuo.*

__Agree / *De acuerdo*
__Neither agree nor disagree / *Ni de acuerdo ni en desacuerdo*
__Disagree / *En desacuerdo*
__Prefer not to answer / *Prefiero no responder*

27. Chocolate clams are an important part of what it means to be a Loretano or to live in this area. *Las almejas chocolatas son una parte importante de lo que significa ser Loretano o vivir en esta area.*

__Agree / *De acuerdo*
__Neither agree nor disagree / *Ni de acuerdo ni en desacuerdo*
__Disagree / *En desacuerdo*
__Prefer not to answer / *Prefiero no responder*

28. Even when I don't use chocolate clams, I like to know they are there. *Aún cuando no uso almejas chocolatas, me gusta saber que estan ahi.*

__Agree / *De acuerdo*
__Neither agree nor disagree / *Ni de acuerdo ni en desacuerdo*
__Disagree / *En desacuerdo*
__Prefer not to answer / *Prefiero no responder*

29. Chocolate clams have value primarily because they provide benefits to people. *Las almejas chocolatas tiene valor principalmente porque proporcianan beneficios a la gente.*

__Agree / *De acuerdo*
__Neither agree nor disagree / *Ni de acuerdo ni en desacuerdo*
__Disagree / *En desacuerdo*
__Prefer not to answer / *Prefiero no responder*

30. Is there anything else you would like to add? *¿Hay algo más que le gustaría decir?*

____
____
____
____

31. Are there any questions I asked you that you'd like to discuss further? *¿Hay algunas preguntas que le pregunté que le gustaría discutir más?*

____
____
____
____
